# JAPANESE OLDER CAREGIVERS’ EXPERIENCES OF PROVIDING CARE AT HOME FOR AN OLDER SPOUSE LIVING WITH DEMENTIA

**DOI:** 10.1093/geroni/igae098.2745

**Published:** 2024-12-31

**Authors:** Mineko Wada, Kazuya Takeda, Hideaki Hanaoka

**Affiliations:** Hiroshima University, Hiroshima, Hiroshima, Japan; Kaneda Hospital, Maniwa, Okayama, Japan; Hiroshima University, Hirishima, Hiroshima, Japan

## Abstract

This qualitative research study explored older caregivers’ experiences of providing home care for an older spouse with dementia in Japan. The proportion of older adults (age 65+) in Japan reached 29% in 2022. The number of households in which one older adult cares for another has been increasing in tandem and is expected to continue to grow steadily. While a growing body of research has focused on the experiences of adult children who provide care for older relatives, little is known about how older adults experience caring for an older spouse at home. In this study, semi-structured individual interviews were conducted with eight older couples in which one partner provided care for their spouse who was living with dementia. Interviews with caregivers were designed to elicit their caregiving experiences and the meanings of caring for their partner at home in their later life. Interviews were digitally recorded, transcribed verbatim, and analyzed thematically. The analysis yielded four themes regarding caregivers’ experiences of older-to-older caregiving: 1) physical burden of caregiving was intensified by caregivers’ physical functioning decline; 2) “I’ll continue to look after my spouse as long as my body’s reached its limit”: a strong sense of love and compassion arising from a long spousal relationship; 3) negotiating the financial burden of caregiving with using formal community care support; and 4) a sense of loneliness: from no family support for caregiving to withdrawal from social networks. We discuss what it means for older caregivers to care for their spouse at home.

